# The IT4 anxiety project : Improving Anxiety Prevention and Management with Innovative Digital Solutions

**DOI:** 10.1192/j.eurpsy.2022.106

**Published:** 2022-09-01

**Authors:** V. De Moffarts

**Affiliations:** Centre Neuro Psychiatrique St Martin, Centre Neuro Psychiatrique Saint Martin, Namur, Belgium

**Keywords:** technology, e-mental health, Anxiety, Europe

## Abstract

Technological innovations give new perspectives in many fields, including health. It was in this context that the IT4Anxiety project was born in 2019 bringing together mental health professionals and start-ups, but also universities, research centres, higher education establishments and public authorities from the North-West Region of Europe. The project is challenging our ability, as partners, to gather stakeholders from different background, medical and non-medical field, in order to support the implementation and co-conception of innovative solutions with the objective of reducing the anxiety of mental health service users. During the four years of project implementation (2019-2023), the stakeholders will have opportunities to work with the end-users, expecting to address the needs of around 3,000 mental health service users suffering from anxiety. The fifteen start-up involved in the project activities will be recruited through hackathons and calls based on identified needs. They will join the project in order to test and improve their solutions. This will create opportunities to connect the start-ups with the medical world, research codes and procedures and to give them a new perspective in the understanding of their targeted market segment. Furthermore, almost a thousand mental health professionals will be trained in e-mental health, benefiting from our training modules and an e-mental health job profile will be designed and implemented in our partner hospitals in Belgium and in Germany.

**Disclosure:**

No significant relationships.

